# The elucidation of the anti-inflammatory mechanism of EMO in rheumatoid arthritis through an integrative approach combining bioinformatics and experimental verification

**DOI:** 10.3389/fphar.2023.1195567

**Published:** 2023-06-01

**Authors:** Pusheng Hui, Sicong Zhou, Chunhao Cao, Wenting Zhao, Li Zeng, Xiaofeng Rong

**Affiliations:** ^1^ Department of Integrated Traditional Chinese and Western Medicine, The First Affiliate Hospital of Chongqing Medical University, Chongqing, China; ^2^ The First Clinical College, Hubei University of Chinese Medicine, Wuhan, China

**Keywords:** Emodin, rheumatoid arthritis, RNA-seq, single cell RNA-seq, inflammatory

## Abstract

**Introduction:** Emodin (EMO), a natural derivative of the anthraquinone family mainly extracted from rhubarb (Rheum palmatum), has previously been demonstrated to possess superior anti-inflammatory properties from a single target or pathway. In order to explore the underlying mechanism of action of EMO against rheumatoid arthritis (RA), a network pharmacology approach was employed.

**Methods:** A gene expression profile from GSE55457 available from the Gene Expression Omnibus (GEO) database was used to identify the targets of EMO action. Further, single cell RNA sequencing data from GEO database of RA patients (GSE159117) were downloaded and analysed. To further investigate the anti-RA effect of EMO on MH7A cells, the expression of IL-6 and IL-1β were monitored. Finally, RNA-seq analyses were conducted on synovial fibroblasts from EMO-treated.

**Result:** We screened the key targets of EMO against RA using network pharmacology methods, including HMGB1, STAT1, EGR1, NR3C1, EGFR, MAPK14, CASP3, CASP1, IL4, IL13, IKBKB and FN1, and their reliability was verified using ROC curve. Single-cell RNA sequencing data analysis showed that these core target proteins mainly played a role by modulating monocytes. The anti-RA effect of EMO was further verified with MH7A cells, which showed that EMO could block cell differentiation and reduce the expression of IL-6 and IL-1β. WB experiments confirmed that EMO could affect the expression of COX2, HMBG1 and the phosphorylation of p38. Finally, sequencing of synovial fibroblasts from rats treated with EMO showed consistent results with those predicted and verified, further proving the anti-inflammatory effect of EMO.

**Conclusion:** Our research shows that EMO inhibits inflammatory response of rheumatoid arthritis (RA) by targeting HMGB1, STAT1, EGR1, NR3C1, EGFR, MAPK14, CASP3, CASP1, IL4, IL13, IKBKB, FN1 and Monocytes/macrophages.

## 1 Introduction

Rheumatoid arthritis (RA) is an autoimmune condition with clinical manifestations including swelling joint pain and stiffness as the main characteristic symptoms (D’Agostino et al., 2016). Approximately 5 out of every 1,000 adults in the world suffer from RA. This disease can occur at all ages, but high prevalence is reported around 60 years old, and the impact on women is 2–3 times that of men (Sparks et al., 2021). Extra-articular features in patients with RA are also very common, and the occurrence of severe infections, respiratory diseases, osteoporosis, cardiovascular diseases, and cancer usually herald adverse outcomes. In the past 20 years, the emergence of biological agents including interleukin 6 (IL-6) inhibitors, costimulatory modulators, B cell depleting drugs as well as tumor necrosis factor (TNF) inhibitors has changed the original treatment strategy and fundamentally Changed the course of rheumatoid arthritis (Crossfield et al., 2021). However, the above-mentioned drugs have varying degrees of adverse reactions or clinical application restrictions, which reduce their expected clinical effects, or they may cause a serious burden on patients‘ medication due to their high price and other factors (Harigai, 2019; Solomon et al., 2020; Tang et al., 2021). Therefore, it is essential to search for more effective, reliable, and cost-effective anti-RA drugs.

Since Chinese medicines are almost natural, they have fewer side effects and lower prices. Some traditional Chinese medicine extracts such as *tripterygium wilfordii* (celastrol) have been proven to have a therapeutic effect on RA in a variety of ways (Song et al., 2020). The plant rhubarb (*rheum palmatum L.*), as one of the traditional Chinese medicines, has a history of thousands of years of use in China. Studies have confirmed that rhubarb and rhubarb extracts have anti-tumor, anti-inflammatory and other pharmacological effect (Shen et al., 2019; Dong et al., 2020). Emodin (EMO) is a natural anthraquinone derivative, which is the active ingredient of rhubarb, Polygonum cuspidatum, Polygonum multiflorum and aloe. It has extensive pharmacological as activities including anti-bacterial, anti-allergic, anti-osteoporosis, anti-viral, anti-diabetic, immune Inhibition, neuroprotection, and liver protection, etc. (Semwal et al., 2021). In an *in vitro* model, EMO exhibits anti-rheumatoid activity through inhibition of the proliferation and metastasis of fibroblast-like synovial cells (Zhu et al., 2019). Although the anti-RA effect of EMO has been demonstrated, the underlying cellular and molecular mechanisms remain unclear. However, due to the limitations of the experimental method, the uncertainty of the research findings is inevitable in the in previous studies. Therefore, in addition to the network pharmacology analysis, RNA sequencing (RNA-seq) and Single-Cell Transcriptome Sequencing analysis were conducted in the present study. It can detect both coding and non-coding genes, enabling a better understanding of potential pathways for disease progression through unbiased analysis (Zhang et al., 2019; K et al., 2021). Finally, we conducted the cell experiments with MH7A *in vitro* to validate the results of systems pharmacology study and RNA-seq.

## 2 Materials and methods

**FIGURE 1:**
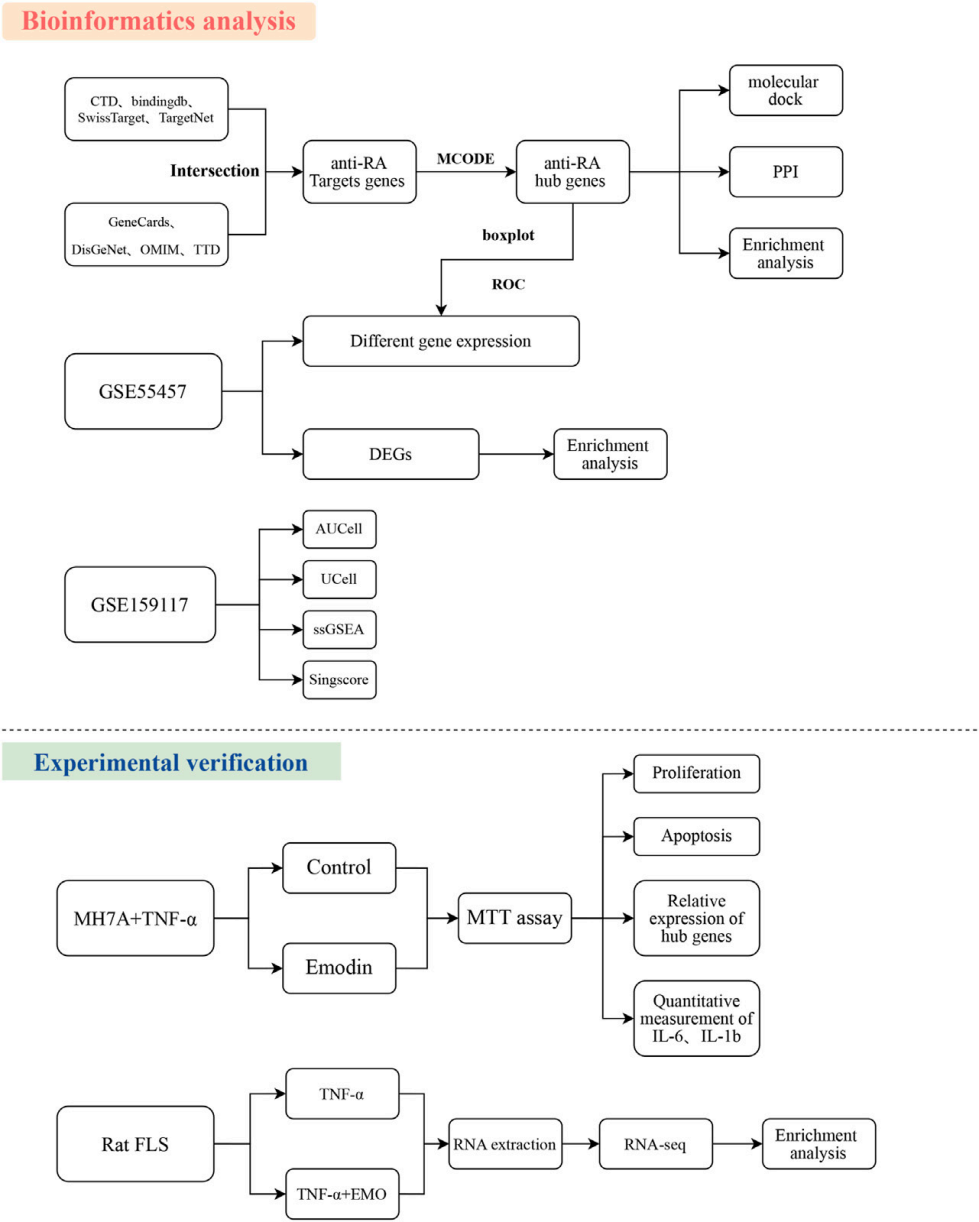
Workflow of the study. The Figure indicates the anti-RA action and mechanism of EMO using the systems pharmacology, RNA-seq, and experimental verification approach.

Flowchart was shown in Figure 1.

### 2.1 Acquisition of EMO pharmacokinetic data

EMO structure was acquired from the NCBI PubChem Database (https://pubchem.ncbi.nlm.nih.gov/) in SMILES format. Absorption, distribution, metabolism, excretion and targets data of EMO were retrieved from CTD (Davis et al., 2021), BindingDB (Gilson et al., 2016), SwissTargetPrediction (Daina et al., 2019), TargetNet database (Yao et al., 2016). All targets were displayed using Venn online tool (Jia et al., 2021).

### 2.2 Targets prediction of EMO against RA

All therapeutic targets of RA were retrieved from OMIM (Amberger et al., 2015) (https://omim.org/, Updated 17 March 2023), DisGeNet (Piñero et al., 2020), TTD database (Wang Y. et al., 2020), and GeneCards (https://www.genecards.org/) using “Rheumatoid Arthritis” as the keyword and “*Homo sapiens*” as the species. Because there are too many targets for RA, we select targets that appear twice or more frequently in the above databases as the targets for follow-up research. A webserver was used for construction of a Venn diagram for visualization of overlapping targets of EMO and RA (https://www.omicstudio.cn/tool/6).

### 2.3 Protein-protein interaction (PPI) network construction

String tool (https://stringdb.org/) was used for topology analysis. RA and EMO common targets were uploaded to String. Then, The MCODE algorithm, a Cytoscape plug-in, is used to identify highly interconnected regions within an interactome, with the genes in these regions being designated as hub genes. (Xie et al., 2022).

### 2.4 KEGG pathway and Gene Ontology enrichment analyses

Functional annotation of the properties of genes and their products in organisms were elucidated through Gene Ontology (GO) analysis (Gene Ontology Consortium, 2015). Differential genes were evaluated through Kyoto Encyclopedia of Genes and Genomes (KEGG) pathway enrichment analysis (Kanehisa et al., 2017). Further, Metascape tool was used perform GO and KEGG enrichment analyses based on the common targets. The results were visualized using ggplot R (version 4.2.2) package. Under the condition of *p* < 0.05, 10 biological processes, cell components, and molecular functions (abbreviated as BP, CC, and MF, accordingly) ranked highest in the GO analysis were extracted. According to the KEGG pathway analysis, EMO is implicated in numerous signaling cascades. Furthermore, the top 20 cascades were picked to construct a barplot.

### 2.5 Molecular docking

The hub genes obtained in the previous step is molecularly docked with the compound of emodin. The molecular docking can effectively determine the small molecule compound that matches the space, force and electrical characteristics 01 the target receptor site and predict the possibility of binding to the target. Firstly, the 3D structures of target proteins were acquired from Protein Data Bank (Berman et al., 2000). AutoDock Vina (Eberhardt et al., 2021) was used to conduct docking of EMO to protein targets and the binding affinity of EMO to protein targets was evaluated by determining the binding energy. The default settings were used and blind docking (entire protein molecule) was conducted.

### 2.6 RNA-seq

#### 2.6.1 Reagents and cell lines

RA-FLS cells (rat) and MH7A cells were acquired from Biovector NTCC (Beijing, CHN). RA-FLS cells were grown incubated at37°C under continuous supply of 5% CO_2_ in DMEM (Cat#11995065, Gibco, United States) enriched with 10% FBS (Gibco, Cat#10099141) and antibiotics, namely streptomycin and penicillin (Cat#15070063, Gibco, United States). To explore the effect of EMO on FLS cells, we divided two different groups. Subsequently, 10 ng/mL TNF-α (Cat#400-14, PeproTechAsia) was administered to the TNF-α group and RA-FLS cells were administered with both TNF-α and 80 μmol/L EMO. After incubating for 24 h, RA-FLS cells were harvested for the following experiment.

#### 2.6.2 RNA extraction

The Qiagen RNeasy Kit (Cat#74106, United States) was used to isolate and purify total RNAs.

The quality of RNA samples was determined using Nanodrop (Thermo Fisher Scientific). AnAgilent 2100 Bioanalyzer was used to evaluate the results.

#### 2.6.3 Preparation of library for transcriptome sequencing (RNA-seq)

In Novogene (China), RNA libraries were prepared, and data was processed. Next, 1 μg of RNA was utilized for each sample, followed by generating libraries using Illumina^®^ NEBNext^®^ UltraTM RNALibrary Prep Kit (NEB, United States), as suggested by the manufacturer. The first step was to purify mRNA by binding polyA to mRNA using OligodT-labeled magnetic beads (llumina Inc., San Diego, United States). The mRNA was then mixed with NEBNext First Strand Synthesis Reaction Buffer (×5). A short fragment of 200–300 bp in size was obtained by mixing. MMuLV Reverse Transcriptase without RNase H activity and random hexamer primers were used to generate first-strand cDNA. Conversion of this cDNA to double-stranded cDNA using RnaseH and DNA Polymerase I. Under the function of 3′-5′ exonuclease and polymerase, fragmentation overhangs were translated to blunt ends. DNA fragments were adenylated, and the fragments were then ligated to the sequencing adapter by A and T complementary base pairing. Then, sequencing libraries were constructed using the AMPure XP system purification method (Beckman Coulter, Shanghai, China) and PCR methods.

Finally, the libraries were evaluated by the Agilent Bioanalyzer 2100 system. Poly (A) selected paired-end sequencing libraries were generated according to the instructions in the TruSeq manual. The sequencer Illumina HiSeq 2000 was emploved to sequence all the libraries.

#### 2.6.4 RNA-seq data analysis

Fastqc (version 0.10.0, Babraham Bioinformatics) was used to perform the quality checks, which can clean up reads by deleting reads that contain adapter/ploy-N and have low-quality. Rat reference genome constructed by paired-end sequencing was aligned against the clean reads using Hisat2 v2.0.5 tool. The number of genes expressed was adjusted to Fragments Per Kilobase of Transcript Sequence per Million mapped reads (FPKM). DEseq (3 biological replicates per condition) was used to identify genes that were differentially expressed genes (DEGs) (*p*-value <0.05). The false discovery rate (FDR) of the *p*-value was normalized using Benjamini-Hochberg method.

### 2.7 Receiver operating characteristic (ROC) curve

In addition, we retrieved GSE55457 from the GEO database to verify the results. The dataset comprised genome-wide transcriptome data from 20 healthy controls, 26 patients with osteoarthritis, and 33 patients with RA. The performance of hub genes was evaluated using the Receiver Operating Characteristic (ROC) curve. For ROC computations, the R package partial ROC (pROC) was employed.

### 2.8 GSEA analysis

We performed GSEA of two groups RNA-seq data by GSEA (R GSVA package, htp://software.broadinstitute org/gsea/index.jsp). Each expression group was classified into downregulated group and upregulated group, and GSEA software was applied to each group for enrichment analysis. This analysis respectively identified the immune related genes that were upregulated or downregulated in RA-FLS. Default weighted enrichment statistics was used for gene enrichment analysis, and the number of replicates was as 1,000. GSEA used FDR to determine enriched terms. FDR <0.25 was used to denote significantly enriched gene sets in GSEA.

### 2.9 Immune infiltration analysis

CIBERSORT (https://cibersort.stanford.edu/) was used to estimate cell type ratios (Zhang et al., 2022). These proportions were then multiplied by a measure of the total fraction in the tissue to produce corresponding estimates. Additionally, combinations of aggregate values were used to estimate the abundance of more comprehensive cell classes, such as lymphocytes, macrophages, and CD4 T cells. Any samples with an estimated *p*-value of <0.05 were reported. Subsequently, the expression differences of hub genes in different immune cells of RA patients and healthy individuals were calculated. The result display is drawn with the pheatmap package in R.

### 2.10 Single-cell RNA-seq

Single-cell mRNA sequencing (scRNA-seq) enables unbiased, high-throughput, and high-resolution transcriptomic analysis of single cells (Kolodziejczyk et al., 2015). We downloaded single-cell transcriptome data from the peripheral blood mononuclear cells of RA patients from the GEO database (GSE159117). Single-cell RNA sequencing (scRNA-seq) data from PBMCs of healthy and RA patients was analyzed using the Seurat (version 4.0) R package. After removing two-cell effects and standardizing, highly variable genes were identified, followed by scaling of all genes using the ScaleData function. Subsequently, thirty statistically significant principal components were chosen as inputs for Stochastic Neighbor Embedding (t-SNE, t-Distributed neighbor embedding). Lastly, classical markers of immune cells were used for annotation, based on previously published literature. To investigate the role of EMO in the scRNA-seq data, the AUCell, UCell, singscore (v1.8.0), and ssgsea modules of the R package were employed (Hänzelmann et al., 2013; Aibar et al., 2017; Bhuva et al., 2020).

### 2.11 *In vitro* cell experiments

#### 2.11.1 Drugs and antibodies

Emodin (purity ≥98%, CAS# 518-82-1) was procured from Shanghai Base Industry (China). Recombinant Human TNF-α(CAT# 300-01A) was purchased from PeproTechAsia (China). Anti-GAPDH (Cat# 5174), COX2 (Cat# #12282), p38 MAPK (Cat# 8690), p-p38MAPK (Cat# 4511) were obtained from Cell Signaling Technology Inc. The HRP conjugated antibody (Goat anti-rabbit, Cat# ab7090) and anti-HMBG1 (Cat# ab18256) antibody was purchased from Abcam Co., LTD., United States.

#### 2.11.2 Cell culture and grouping

After thawing and resuscitating, MH7A cells were inoculated in DMEM medium containing high level of glucose medium supplemented with FBS (15%), and antibiotics (100 U/mL penicillin, and 100 μg/mL streptomycin). A cell culture incubator (Sanyo, Japan) was used to culture the cells at 5% CO2 and 37°C. Fresh medium was used to replace the culture medium every 2 days. When the confluence of cells reached 85%, trypsin digestion and passage to the fourth generation at a ratio of 1:3 for follow-up experiments. Five experimental groups were used in the current study, namely the control group (untreated), the TNF-α group (cells were administered with TNF-α at a concentration of 10 ng/mL), and the 20 μmol/L EMO group (exposed to 20 μmol/L EMO and 10 ng/mL TNF-α), 40 μmol/L EMO group (given 10 ng/mL TNF-α and 40 μmol/L EMO treatment) and 80 μmol/L EMO group (co-administered with 80 μmol/L EMO and 10 ng/mL TNF-α). Analysis of each group was conducted in triplicates.

#### 2.11.3 MTT assay

MH7A cells at a density of 5,000 cells per well were grown in a 96-well plate after attaining the logarithmic growth phase. The plate contained complete medium, and cells were cultured overnight in an incubator. According to different groups, the corresponding drugs were given intervention for 24 h, and 6 parallel holes were set for each treatment. After the intervention, the culture plate was taken out, the culture medium was aspirated, and 20 μL of MTT reagent (Cat# C0009S, Beyotime, China) at 5 g/L concentration was mixed with MH7A cells and incubation was conducted for 4 h. MTT crystals were mixed with 200 μL of dimethyl sulfoxide. Subsequently, a microplate reader (Thermo Fisher, the United States) was used to read the optical density value (OD value) of the cells in each treatment group at 450 nm. In each group, the survival rate of cells was evaluated using the underlined formula; cell survival rate = (EMO group OD value/the control group OD value) ×1 00%. The experiment was conducted in triplicates.

#### 2.11.4 Wound healing assay

The effect of water level on the migration of MH7A cells was assessed by wound healing assay. FLS (1 × 10^5^) of MH7A cells with or without TNF-α were seeded into six-well plates and a uniform scratch was made with a sterile 10 μL tip. Images of cells migrating into the wound were captured at 0 and 24 h using inverted microscope (×100) and the area of the scratched zone was measured by ImageJ (Version 1.52) analysis system.

#### 2.11.5 Cell cycle

The cell cycle was identified using flow cytometry. MH7A cells in logarithmic growth phase at a cell density of 1 × 10^8^/L were transferred to a 6-well plate with 3 mL per well. After 24 h of incubation in a constant temperature CO_2_ incubator, the cells were collected from each group, rinsed twice with pre-cooled 0.01 mol/L PBS (pH 7.2), and fixed overnight with 75% cold ethanol at 4°C. Removing the fixative before testing, add 300 μL of cell cycle staining solution for 30 min, vortexing to disperse the cells (2 s each time, 10 times in total) and ice-bath for 2 h in the dark. Taking 3 × 10^4^ cells for each group for Cytometer detection. FLOW JO (version 10.6.2) software was used to analyze the phase ratio and early cell apoptosis rate at each time of the cell cycle. The experiment was repeated three time.

#### 2.11.6 ELISA enzyme-linked immunosorbent assay

Levels of IL-1 β (Cat# KE00021, Proteintech, United States) and IL-6(Cat# EK0410, BOSTER, China) in the cell supernatant were determined by ELISA. The well-growing MH7A cells were grown into a 6-well cell plate containing high-sugar medium at 3 × 10^5^ cells in each well, and then incubated. According to the grouping administration treatment under “5.7.3”, each group is provided with three repetitive holes. After 24 h of treatment, the supernatants of each treatment group were collected, and IL-6 and IL-1β contents were evaluated by ELISA kit. ELISA experiment was conducted in triplicates.

#### 2.11.7 RT-PCR

MH7A cell were incubated with 80*M EMO for 24 h. RNA was then extracted from the cells with a Takara RNA Extraction Kit (Cat# 9767, Japan), following the instructions given by the manufacturer. RNA was subjected to reverse transcription to obtain cDNA. RT-PCR was conducted using the TB Green qPCR kit (Cat# RR420A, Takara, Japan) following the 20 ructions provided by the manufacture. In the PCR method, initialization was performed at 95°C for 5 min, then denatured at 95°C for 39 cycles and a period of 30 s, 15 s of annelation at 55°C, and further extended for 30 sat 72°C. The 2^−ΔΔCt^ method was used for analysis of 19 expression data. Primer sequences applied in RT-PCR are presented in Table 1.

**TABLE 1:** RT-PCR primer sequences.

Primer name	Primer sequences (5′to 3′)
PTGS2-R	AAG​ACA​GAT​CAT​AAG​CGA​GGG​C
PTGS2-F	AAA​CCG​TAG​ATG​CTC​AGG​GAC​T
GAPDH-F	GGA​AGC​TTG​TCA​TCA​ATG​GAA​ATC
GAPDH-R	TGA​TGA​CCC​TTT​TGG​CTC​CC

#### 2.11.8 Western blot analysis

The Nuclear and Cytoplasmic Protein Extraction Kit (Cat# P0028, Beyotime, China) was used to extract proteins from cells. Taking 60 μg of denatured protein for SDS-PAGE separation, the electroporation was then transferred to PVDF membrane and immersed in fresh TBST solution containing 5% skimmed milk powder for 1 h. The membrane was then incubated with primary antibody diluted 1,000 times in TBST solution at 4°C for 24 h. Following this, the membrane was washed with TBST solution three times, 10 min/time, before being incubated with secondary antibody diluted 5,000 times in TBST solution at 37°C for 1 h. Upon adding a chemiluminescent agent to develop and expose in the dark, the gel imaging system of Bio-Rad Company of the United States was used for scanning and analysis. The intensity of each protein was normalized with GAPDH, and the experiment was repeated three times.

### 2.12 Statistical analysis

GraphPad Pris 7.0 and R software were used for analysis of data and generation of figures and plots. The present data were presented as mean ± standard error of the mean (SEM) of triplicate experiments. Differences between groups were explored using One-way analysis of variance ANOVA) then pair-wise differences between groups were determined by conducting Tukey’s *post hoc* test. *p*-value <0.05 denoted that the differences between groups were statistically significant.

## 3 Results

### 3.1 Putative targets of EMO and RA using network pharmacology-based prediction

**FIGURE 2:**
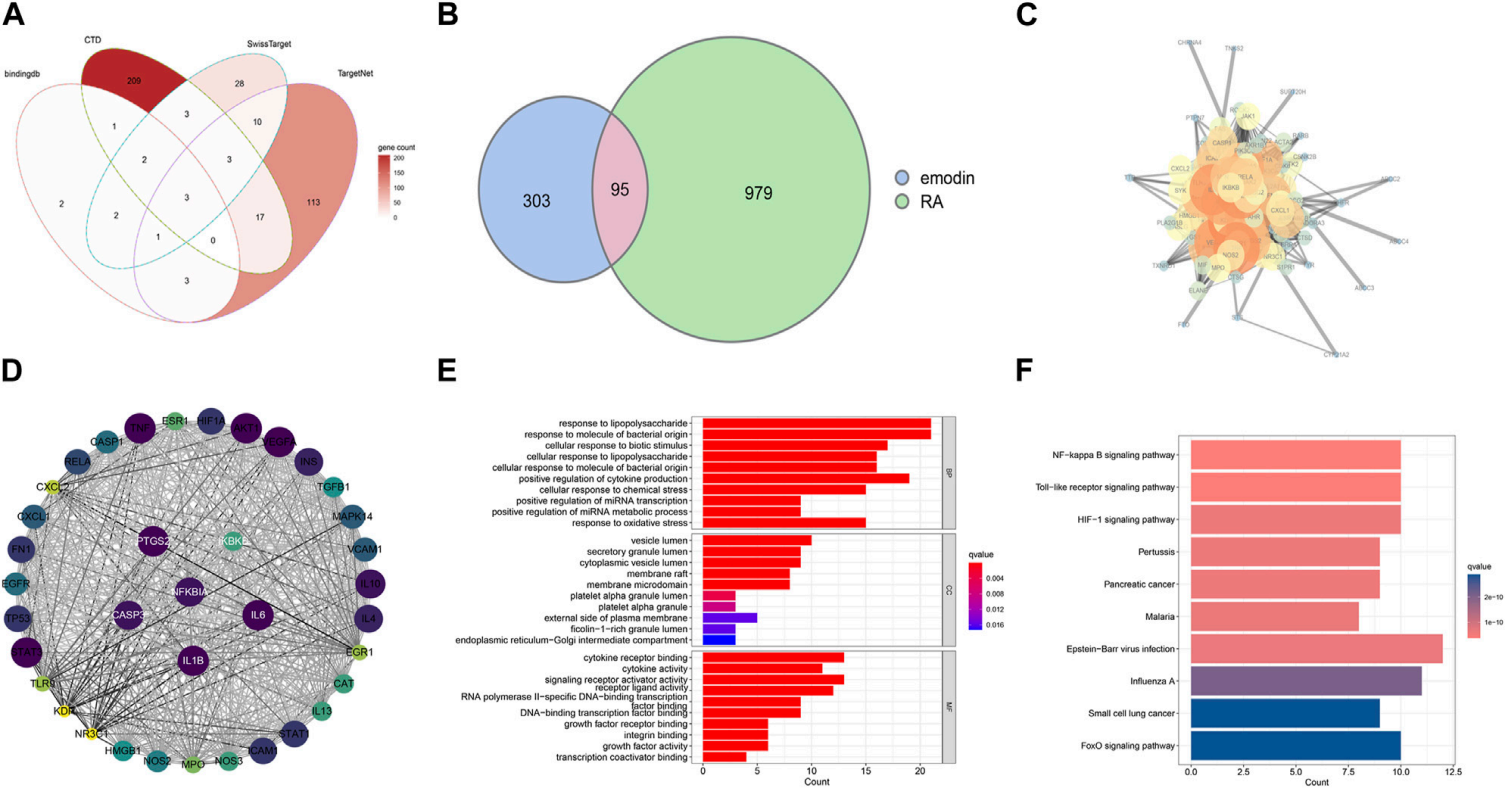
Targets of EMO and RA. **(A)** EMO targets in four databases; **(B)** A Venn diagram showing and RA therapeutic targets and putative EMO targets; the pink area represents the common target of emodin and RA; **(C)** A PPI network showing potential EMO targets. Proteins are indicated by network nodes whereas edges represent protein-protein interactions. **(D)** The MCODE plugin screened 37 hub genes; Enriched GO terms **(E)** and Enriched KEGG pathways **(F)** related with potential targets of EMO against RA.

Following removal of duplicates, a total of 398 EMO-target genes were used for subsequent analysis (Figure 2A). Enrichment analysis of these 398 genes revealed that they were predominantly associated with apoptosis, IL-17 signaling pathway, and T cell receptor signaling pathway (Supplementary Figures S1A, B). Additionally, using OMIM, DisGeNET, TTD and GeneCards, a set of 1079 RA-related genes were obtained, and the results of their enrichment analysis showed considerable overlap between the top 20 RA-related pathways and the mechanism of action of EMO (Supplementary Figures S2A, B). Furthermore, 95 overlapping therapeutic targets between EMO and RA were visualized using Venn software (Figure 2B). The PPI network of the assumed target of EMO-RA was constructed using the STRING platform and visualized in Cytoscape 3.7.1 (Figure 2C). The MCODE plug-in was used to obtain 37 hub genes (Figure 2D). Enrichment analysis of these hub genes showed that, based on the network pharmacological prediction method, the anti-RA effect of EMO was closely related to cytokine receptor binding, with NF-kappa B as the main enrichment pathway (Figures 2E, F).

### 3.2 Verify the reliability of hub genes

**FIGURE 3:**
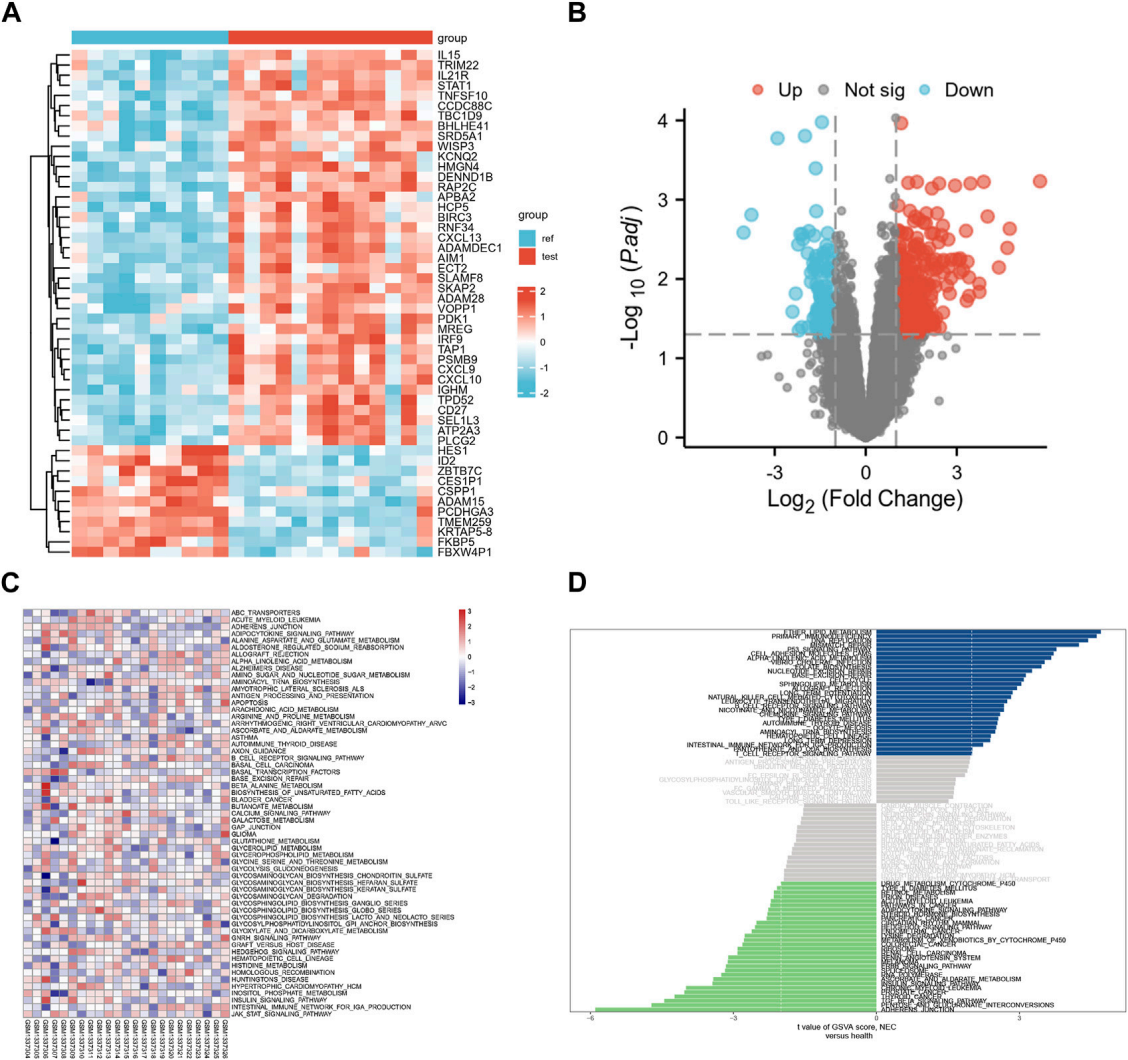
**(**A**)** Heatmap of differential gene expression, with blue representing healthy individuals and red representing RA patients; **(B)** Differential gene volcano map, red represents upregulated differential genes, blue represents downregulated differential genes; **(C)** GO enrichment analysis map; **(D)** Barplot of KEGG pathway enrichment analysis.

**FIGURE 4:**
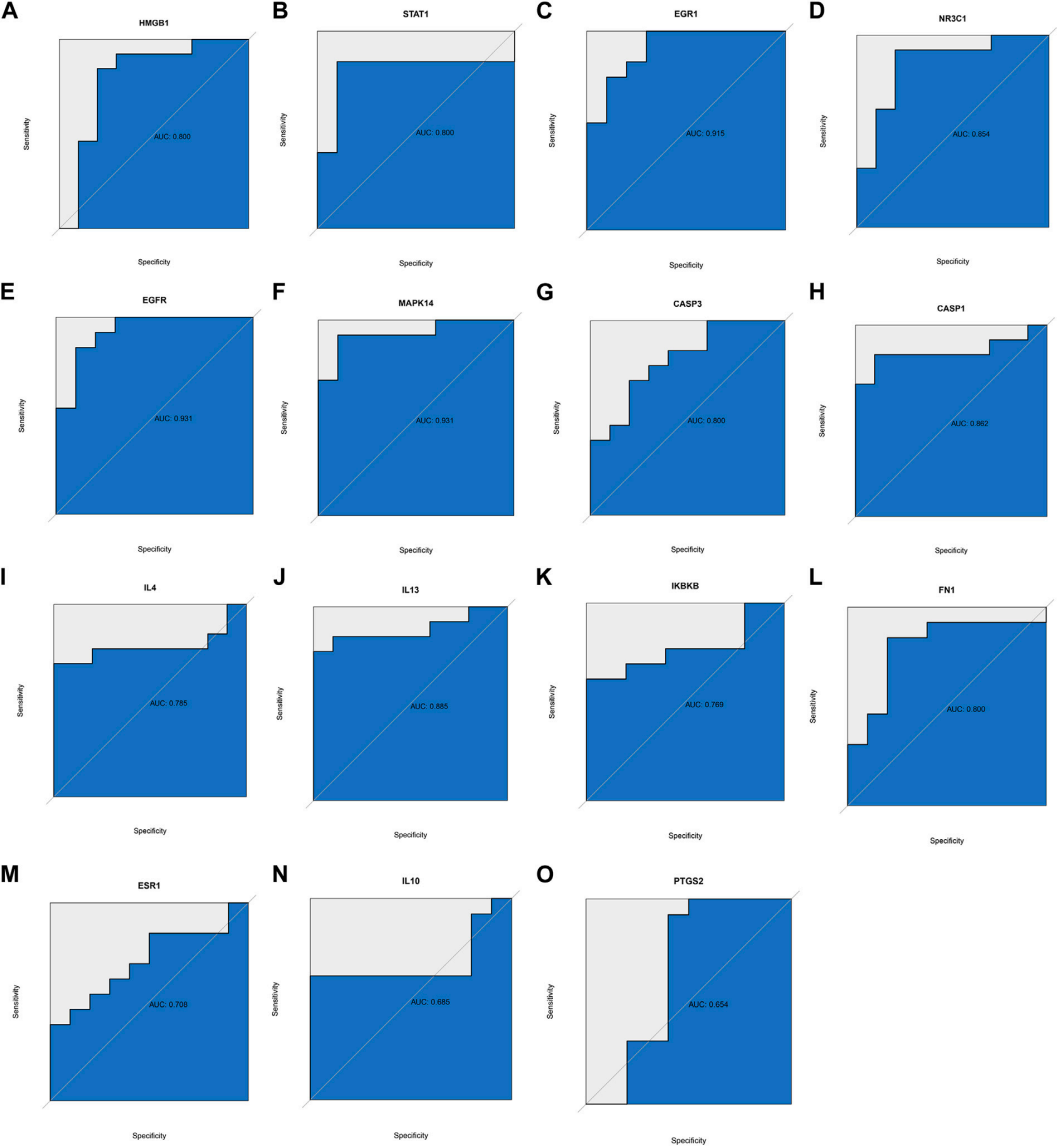
ROC curve was used to verify whether hub genes could distinguish RA patients from healthy patients, based on the GSE55457 dataset. **(A)** HMBG1, **(B)** STAT1, **(C)** EGR1, **(D)** NR3C1, **(E)** EGFR, **(F)** MAPK14, **(G)** CASP3, **(H)** CASP1, **(I)** IL4, **(J)** IL13, **(K)** IKBKB, **(L)** FN1, **(M)** ESR1, **(N)** IL10, **(O)** PTGS2.

Given that RA targets obtained from the database may differ from reality, we downloaded transcriptome data sets of RA and healthy human synovium samples from the GEO database. We used the R language to visualize the distribution of differential genes in gene sets (Figures 3A, B). These differential genes belonged to inflammation-related factors and chemokine-related factors, among others. To further verify the key biological processes and pathways of RA pathogenesis, we conducted GSVA analysis (Figures 3C, D). Finally, our ROC curve confirmed that 15 genes could serve as effective classifiers (Figure 4, Only genes with AUC greater than 0.65 were selected). These 15 genes (HMGB1, STAT1, EGR1, NR3C1, EGFR, MAPK14, CASP3, CASP1, IL4, IL13, IKBKB, FN1) are considered to be the key genes for the anti-RA effect of EMO.

### 3.3 Immune infiltration analysis

**FIGURE 5:**
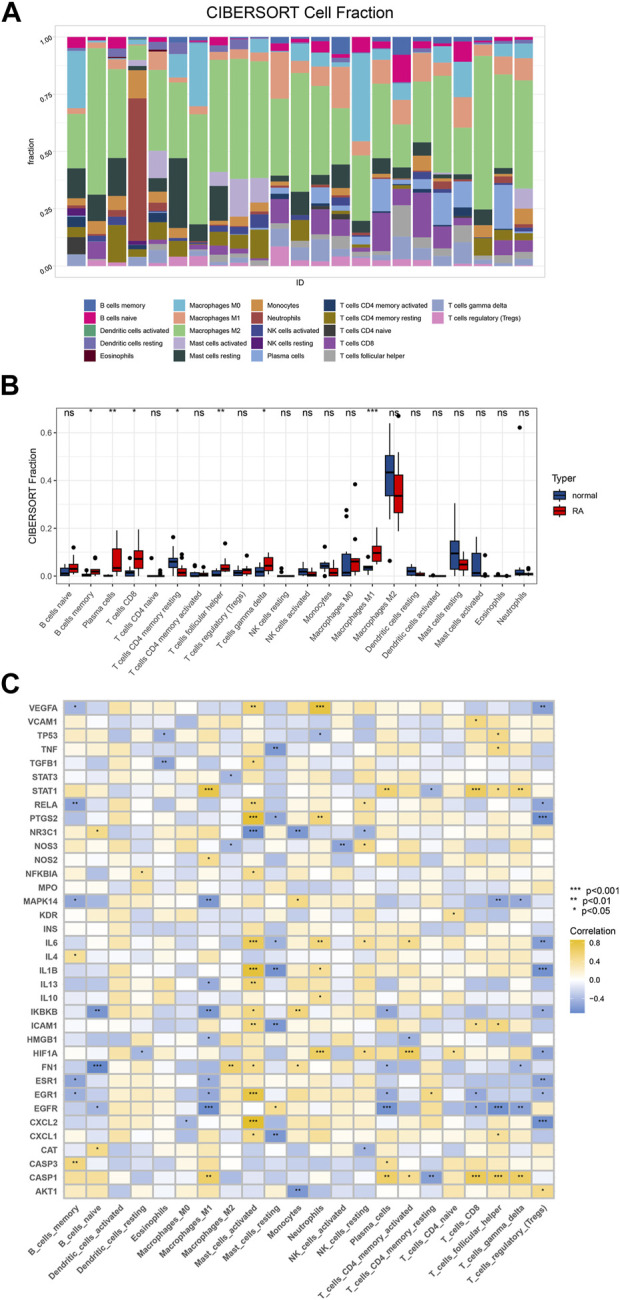
Immunoinfiltration analysis. **(A)** Based on CIBERSORT algorithm, the immune cell infiltration of RA samples and control samples in GSE55457 was analyzed, and the proportion of immune cells in each sample was analyzed; **(B)** To compare 22 kinds of immune cells between groups and draw a boxplot of different cell types; **(C)** Correlation analysis between hub gene and immune cells.

Among the 22 immune cell types inferred by CIBERSORT, the components of M1 macrophages, plasma cells, and follicular helper T cells were differentially expressed between RA and normal patients (Figures 5A, B). To explore the differences in the expression of the selected hub genes in various immune cells, we used R language to draw correlation heat maps of the data. As expected, our analysis showed statistically significant differences in the expression of the selected hub genes in macrophages (Figure 5C).

### 3.4 Single cell RNA sequencing analysis

**FIGURE 6:**
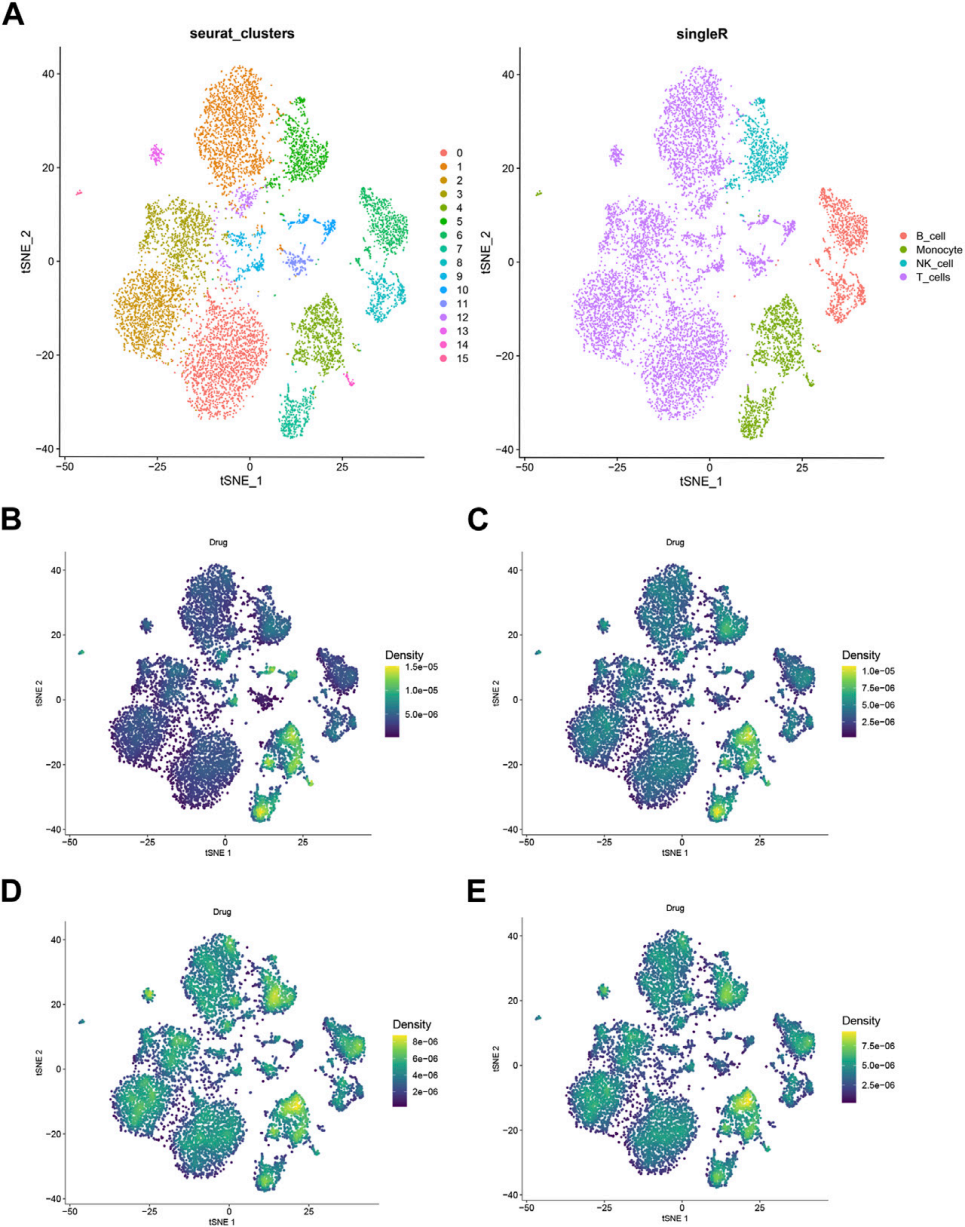
Single-cell RNA sequencing analyzed. **(A)** TSNE plots of 23 subgroups **(B)** hub gene singscore scores of each cell sample in the GSE159117 dataset. **(C)** Hub gene ssgsea score for each cell sample in a single cell dataset; **(D)** hub gene AUCell scores of each cell sample in GSE159117 dataset. **(E)** Hub gene Ucell scores for each cell sample in the GSE159117 dataset.

Analysis of the hub gene sets of EMO drug clusters was conducted using AUCell, Ucell, ssgsea, and singscore algorithms. The results demonstrated that the chosen hub genes had a significant effect on monocytes, as shown in Figure 6, regardless of the algorithm used.

### 3.5 Molecular docking

**FIGURE 7:**
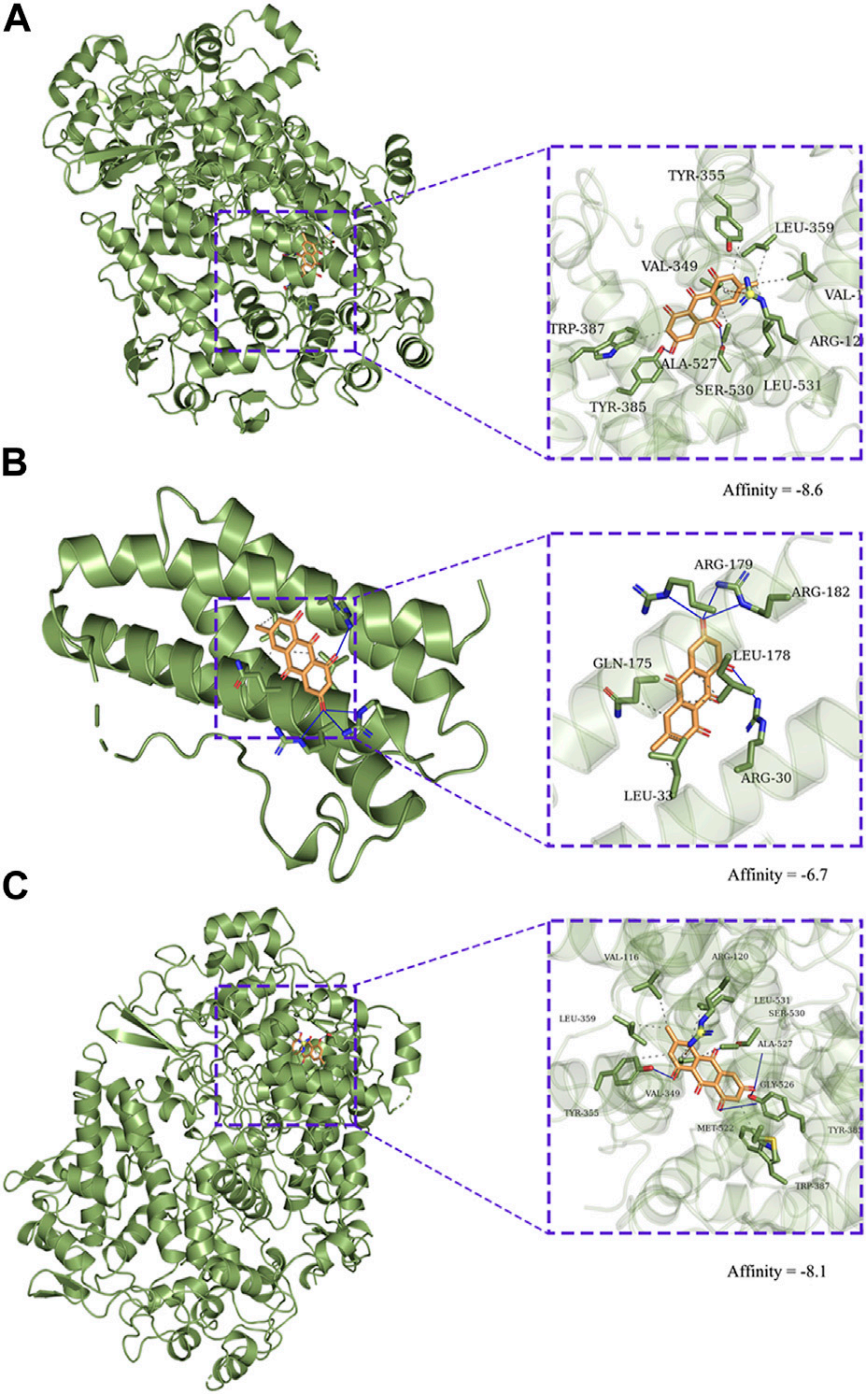
Molecular docking diagram of EMO to targets. Zoom (left) and focus (right). **(A)** CASP3, **(B)** IL6, **(C)** PTGS2.

EMO was docked with the core target proteins MAPK14 and PTGS2 the docking scores were indicated in Figure 7. The output optimization of the results using AutoDock Vina software was shown in Figure 7. The lower binding energy suggests that the target protein and EMO have a strong interaction. The results showed that the target proteins MAPK14, IL6, PTGS2 had strong binding energy with EMO.

### 3.6 Variations in the mRNA expression genes in rat RA-FLS post TNF-α administration and EMO treatment

**FIGURE 8:**
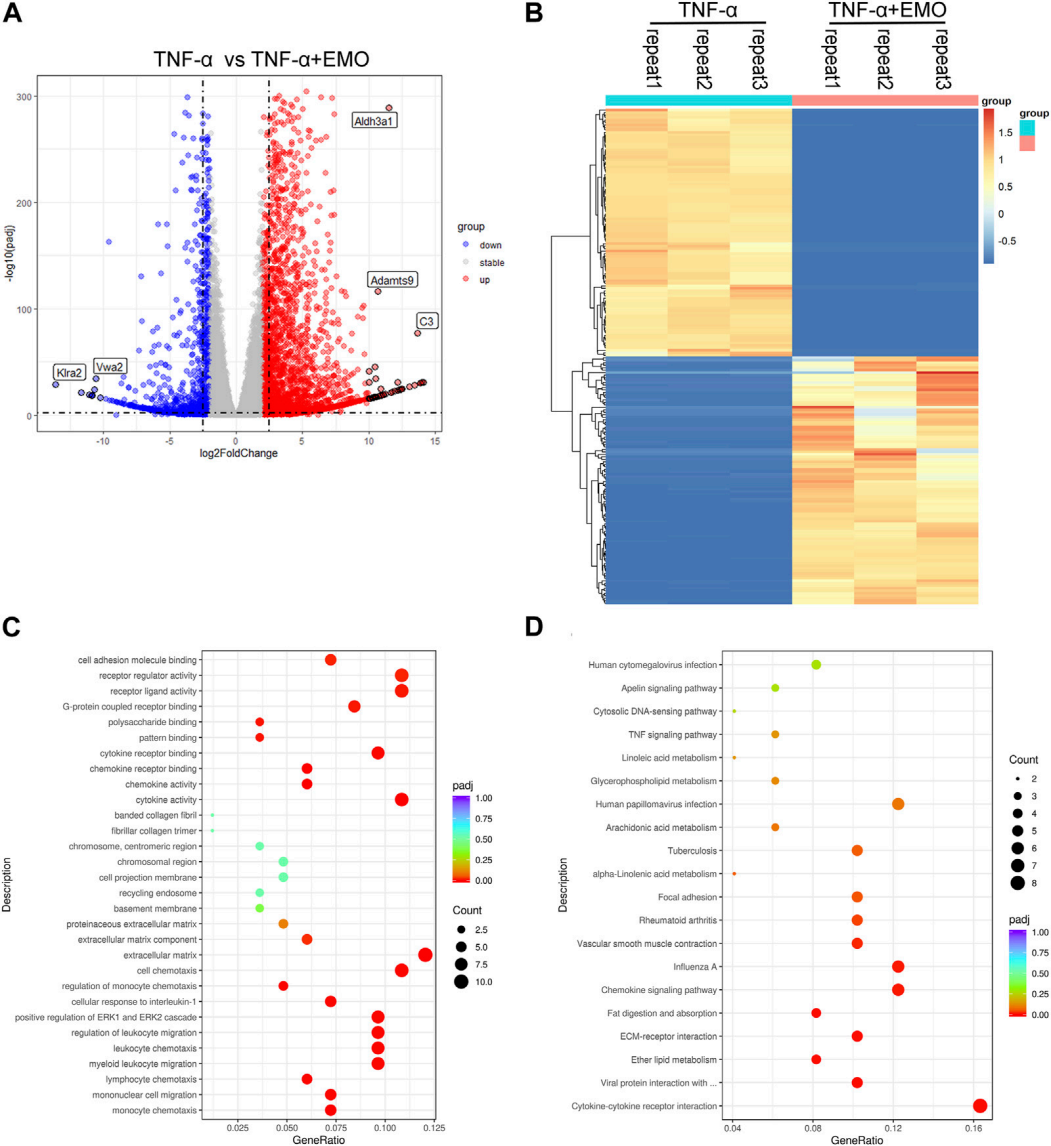
Analysis of rat RA-FLS gene expression profiling after TNF-α administration and EMO treatment. **(A)** Volcano plot indicating the mRNA levels of DEGs. **(B)** A heatmap showing mRNA expression levels of DEGs. DEGs were grouped into two classes, upregulated and downregulated genes (shown in red and blue color, accordingly). The dark color indicates the most significant variations. **(C)** The top 10 markedly enriched MF, BP, CC GO terms related to the DEGs. **(D)** The top 20 significantly enriched pathways.

To study EMO function on RA, we established *in vitro* RA model on rat RA-FLS, which was triggered using TNF-α(10 ng/mL). The results showed that 80 μmol/L EMO exhibited anti-proliferative activity against RA-FLS. The mechanisms of EMO inhibitory action on RA-FLS proliferation induced by TNF-α were then investigated using RNA-seq. The obtained data indicated significant changes in gene expression profiles in the EMO-treated group (TNF-α+EMO) and the non-EMO-treated group (TNF-α). In the present study, 3385 DEGs were obtained in RA-FLS after administration of TNF-α using with a fold change >2 and *p*-value < 0.05 as the settings (Figures 8A, B). Moreover, KEGG analysis indicated that 153 cascades were enriched, mainly chemokine signaling pathway, ECM-receptor interaction and cytokine-cytokine receptor interaction (Figures 8C, D).

The data reported in this study have been uploaded in the GEO database (GSE232679).

### 3.7 EMO modulates proliferation of MH7A in a dose-dependent manner

**FIGURE 9:**
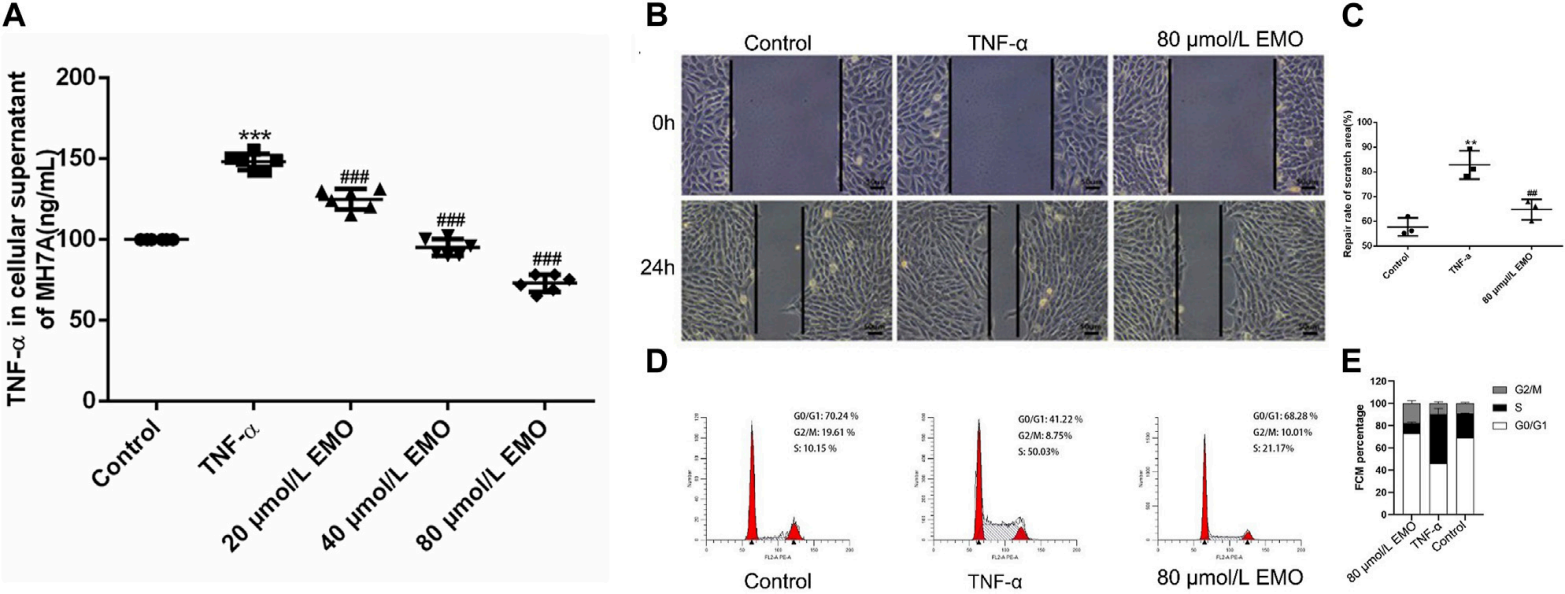
Effects of EMO on migration and proliferation. **(A)** Three different concentrations of EMO on the proliferation of MH7A. EMO inhibited the proliferation of MH7A, and this effect was dose. Dependent. The results were indicated as the means ± SD. In the figure, represents *p* < 0.001 relative to untreated group whereas, ^##^ indicates *p* < 0.001 relative to the TNF-α treatment group. **(B)** A representative image of Transwell. **(C)** Number of migrated cells in the TNF-α and EMO groups in the MH7A cells. SEM is present as error bars in the figure Data are presented as mean from triplicate experiments. ^*^Denotes *p* < 0.05, ^**^represents *p* < 0.01 and ^***^represents *p* < 0.001. **(D)** A representative image of the cell cycle analyzed by flow cytometry. **(E)** FCM percentage in the different groups was shown, ^***^represents *p* < 0.001 relative to the control group. ## indicates *p* < 0.001 relative to the TNF-α group.

MTT test results revealed that the survival rate of cells in the TNF-α group was markedly higher relative to the survival rate of cells in the untreated group (Figure 9A). In addition, cells in the 20, 40, and 80 μmol/L EMO treatment groups showed significantly lower survival rates relative to the survival rates of cells treated with TNF-α (Figure 9A). The findings revealed that EMO inhibited proliferation of MH7A with higher inhibition observed for the 80 μmol/L EMO group. According to the results of MTT, we selected the dose concentration of the group with the lowest MH7A cells survival rate as the EMO concentration for subsequent experiments. Thus, the subsequent experiments were divided into three groups: control, TNF-α, and 80 μmol/L EMO.

### 3.8 EMO inhibits TNF-α induced migration and proliferation of MH7A cells

In the effect of EMO on invasion of MH7A cells was explored through a transwell assay. MH7A cells that moved to the lower chamber were markedly more in the TNF-α group re lative to cells that migrated after treatment with EMO (Figures 9B, C). This implies that EMO significantly suppressed the cell migration ability of MH7A cells (*p* < 0.05). Flow cytometry revealed that EMO had a significant effect on the cell cycle of MH7A cells, with statistical significance in the proportion of S phase between the three experimental groups (Figure 9D, *p* < 0.05). This was evidenced in Figure 9E.

### 3.9 EMO treatment induced differential expression of hub genes in MH7A cells

**FIGURE 10:**
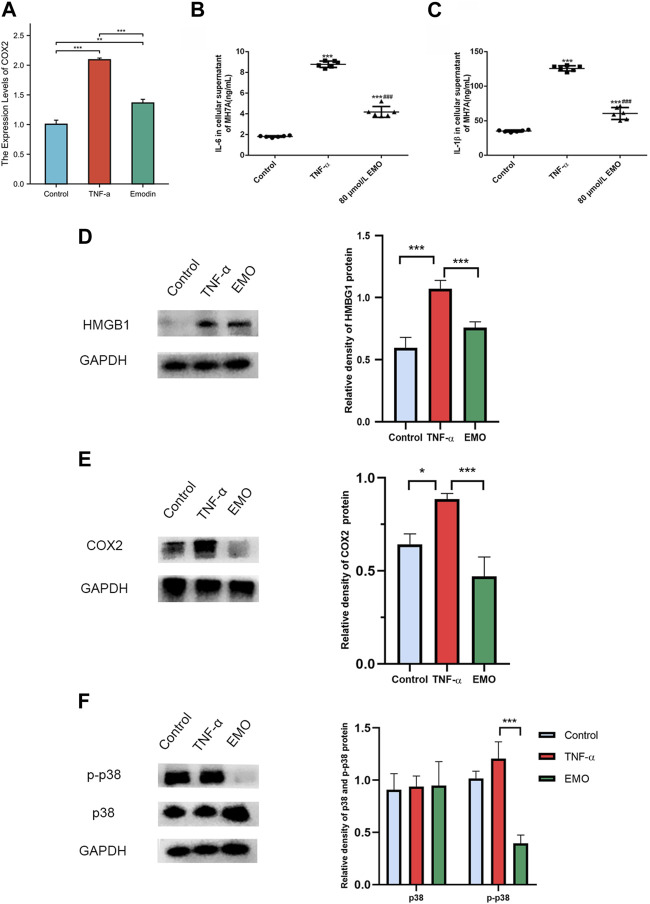
EMO alleviates inflammation in MH7A cells. **(A)** RT-PCR analysis of COX2 expression levels in EMO-treated MH7A cells. ELISA was conducted to measure the content of cytokine in cell culture supernatant. IL-6 **(B)** and **(C)** IL-1β levels were lower in cells of the 80 umol/L group relative to the levels in cells of the TNF-α group. ^***^ denotes *p* < 0.01 relative to the control group. ^###^ denotes *p* < 0.001 relative to content in TNF-α group. The expressions of HMGB1 **(D)** and COX2 **(E)** proteins were significantly decreased in EMO group. **(F)** EMO significantly reduced the phosphorylation level of p38MAPK.

The effect of EMO treatment on expression of hub genes in MH7A cells was further verified by rt-PCR analysis. The expression of inflammation-related genes PTGS2 (COX-2) was significantly downregulated in the EMO group relative to the control group (Figure 10A).

### 3.10 Effects of EMO on IL-1β content and IL-6 levels in MH7A cells

IL-1β and IL-6 contents in the supernatant of MH7A cells in the TNF-α group were markedly higher compared with the contents in the control group (*p* < 0.05). Notably, treatment of MH7A cells with 80 μmol/L EMO after treatment with TNF-α markedly reduced IL-1β and IL-6 contents relative to levels of the two cytokines in the control group (Figures 10B, C).

### 3.11 EMO inhibited invasion by attenuating HMGB1 and COX2 expression and phosphorylation of p38

Compared with the control group and TNF-α group, HMBG1 and COX2 proteins in the EMO group showed a clear decrease in expression (Figures 10D, E). Furthermore, as anticipated, the MAPK pathway was significantly affected by EMO. Phosphorylation of P38MAPK was inhibited in the presence of EMO, while expression of p38MAPK did not differ significantly between the three groups (Figure 10F). Overall, these results suggest that EMO modulates the inflammatory response induced in MH7A cells.

## 4 Discussion

As the main component of rhubarb, EMO is an anthraquinone compound that has anti-inflammatory, immunomodulatory, anti-viral and anti-tumor pharmacological effects (Semwal et al., 2021). Studies have confirmed that EMO can attenuates acute pancreatitis-associated lung injury by reducing pancreatic exosome production and altering exosomal protein contents (Hu et al., 2022); in addition, in the microglia stimulated by LPS, it can also play an anti-neuro- inflammation effect via AMPK/Nrf2 signal pathway (Park et al., 2016). A study by Mengmeng Zhu demonstrated that emodin improves rheumatoid arthritis by promoting neutrophil apoptosis (Zhu et al., 2019). However, it is becoming increasingly recognized that Chinese herbal medicine exerts pharmacological effects through targeting a variety of proteins (Li et al., 2019; Wang J. et al., 2020). To explore the mechanism of action of EMO, we employed network pharmacology. Previous TCM related network pharmacology studies, however, only predicted drug action targets through databases, which may not be completely consistent with the actual target of EMO action. To overcome these shortcomings, we applied RNA-Seq and RNA-Seq in this study, providing more convincing evidence. Our results indicate that the hub genes of the anti-Ra effect of EMO are CASP1, CASP3, EGFR, EGR1, FN1, etc. According to network pharmacology analysis results, the action pathways of EMO were found to be mainly related to inflammation, infection, and cell fate, particularly the macrophage. We also focused on the influence of EMO on the cytokines IL-6 and IL-1β, which are secreted mainly by macrophages (Yao et al., 2014; Rodriguez et al., 2019). We then conducted a series of experiments to confirm our findings, which demonstrated that EMO effectively inhibited the proliferation of RA-FLS cells.

In recent years, it has been found that abnormal cell apoptosis can lead to excessive hyperplasia and hypertrophy of synovial tissue, thereby promoting the occurrence and development of RA (Misra et al., 2021). Synovial cells of RA patients have abnormal apoptosis process. Abnormal cell apoptosis in RA is not only related to the abnormal expression of apoptosis-related genes, but also many molecules are involved in the regulation of apoptosis, such as Fas-gene, Bcl-2 gene and some oncogene and Caspase-3, etc., (Zhu et al., 2016; Ma et al., 2021). Caspase-3, also known as cysteine protease 32, is one of the most important executors of apoptosis in the Caspases family. There is evidence that proteases are closely related to apoptosis, which seems to be a process of protease cascade cleavage. Different proteases respectively cleave the pre-Caspase-3 thereby activating Caspase-3. The activated Caspase-3 cleaves different substrates (including cytoskeleton proteins and DNA repair proteins, etc.), resulting in protease cascade cleavage and amplification, and finally the cell moves towards apoptosis. Studies have confirmed that Caspase-3 expression is upregulated and the level of apoptotic cells in the synovial tissue of rats treated with moxibustion is relatively high, and the symptoms of RA are alleviated. However, apoptosis is not activated by a single pathway, but by many parallel pathways, when one of the pathways is blocked, it cannot completely inhibit apoptosis (Lakhani et al., 2006, 7). It is still necessary to conduct in-depth research on the various pathways that cause apoptosis, and to find out the key points of the apoptosis pathway, to open up new ways for the treatment of RA.

PTGS2 is the core gene in the biosynthesis of prostaglandin, which is responsible for the prostanoid biosynthesis involved in inflammatory response and regulation of the content of inflammatory cytokines (Bellone et al., 2006; Li et al., 2020). Among the inflammatory cytokines related to RA, IL-1 and IL-2 are mostly studied. Existing studies have found that after RA modeling in rats, the content of IL-1 in the blood increases, and the content of IL-2 decreases. The content of IL-1 in rats that received moxibustion acupuncture and infrared treatment was significantly downregulated relative to that of animals in the RA model group. Moreover, the content of IL-2 of rats that had undergone moxibustion acupuncture and in frared treatment was upregulated relative to that of rats in the RA model group. In addition, chemicals released during inflammation such as serotonin, prostaglandins, and cytokines including TNF-α can activate pain receptors. TNF-α is also an important inflammatory factor that mediates synovitis and the breakdown of the junction between bone and cartilage (Zhang et al., 2021). ESR1 (ERa), which is key to estrogen receptor regulation, is expressed in osteoblasts, osteoclasts, and chondrocytes (Dall and Britt, 2017; Lu and Herndon, 2017). After combining with the nuclear estrogen receptor. Estrogen exerts its biological effects through various cascade reactions. In recent years, several clinical observational programs have confirmed that estrogen has a dramatic effect on RA, because it can be found in the clinic that the incidence of RA in menopausal women is significantly higher than that in men of the same age, and the incidence is basically the same for men and women after the age of 80; RA patients often relieve spontaneously during pregnancy, and may worsen after childbirth (Sapir-Koren and Livshits, 2016).

HMGB1 has been demonstrated to play an essential role in autoimmune diseases such as rheumatoid arthritis (RA), as well as in inflammatory events (Zhou et al., 2008). Specifically, HMGB1 has been shown to induce the release of TNF-α from activated macrophages, which in turn leads to macrophage translocation to the cytoplasm. Furthermore, HMGB1 has been found to promote osteoclast formation by binding to a receptor activator of NF-κB ligand, thus inducing additional destructive effects on the joint tissues of RA patients (Zhou et al., 2008). Notably, NF-κB-related genes were identified as a core target of EMO.

NF-κB signaling pathway is extensively involved in non-specific immune processes and inflammatory responses, and the proteins implicated in this pathway include RelB, RelA/P65, NF-κB1 P50/P105, C-REL and NF-κB2 P52/P100. Activation of this pathway activates the expression of immune molecules, producing a powerful inflammatory response (Ghosh et al., 1998). In general, NF-κB binds to its inhibitory protein IκB and does not play a biological role, however, in the presence of activators including IL-17, TNF-α as well as IκB undergo phosphorylation and ubiquitination, and finally broken down by enzymes under the action of IκB kinases (IKKs). After breaking down of IkB, the nuclear entry signal of transcription factor NF-κB is exposed and immediately enters the nucleus to begin the transcription process (Baldwin, 1996). Activated NF-κB signaling pathway induces T cell activation, mediates inflammatory response, and leads to abnormal proliferation of RA-FLS, which stimulates osteoclast proliferation, joint deformity, and bone erosion, thus exacerbating RA. In addition, activation of NF-κB produces cytokines and acute response proteins that activate the immune response and increase the expression of adhesion molecules, which increase the inflammatory level of RA. Moreover, immune products (IL-1β, TNF-α, IL-17, IL-6, etc.) produced by NF-κB signaling pathway can activate the NF-κB signaling pathway, this positive feedback effect keeps the NF-κB signaling pathway activated, which can be terminated when the NF-κB signaling pathway is inhibited. In addition, existing studies also confirm that chondrocytes are implicated in progression of RA by modulating activation of NF-κB mediated by transmembrane protein 147 (TMEM147) (Ota et al., 2020). Studies should thus be conducted to design drugs that inhibit this pathway in RA treatment.

In summary, our study revealed that EMO holds an anti-inflammatory effect for RA, targeting HMGB1, STAT1, EGR1, NR3C1, EGFR, MAPK14, CASP3, CASP1, IL4, IL13, IKBKB, FN1. Though promising, there are still areas of improvement. The RNA-seq data was sourced from synovial cells while the sc-RNA-seq was sourced from peripheral blood mononuclear cells, exposing potential differences. Moving forward, our research group will conduct animal experiments to validate EMO’s role *in vitro* and conduct single-cell RNA sequencing of synovial cells to further explore the local targets of EMO in the joint.

## Data Availability

The datasets generated or analyzed for this study can be found in the GSE232679, GSE55457, GSE159117 https://www.ncbi.nlm.nih.gov/gds.
