# Supplementary figures and images for: The elucidation of the anti-inflammatory mechanism of EMO in rheumatoid arthritis through an integrative approach combining bioinformatics and experimental verification
Source: Front Pharmacol. 2023 Jun 1;14:1195567. doi: 10.3389/fphar.2023.1195567 (PMC10267444; doi:10.3389/fphar.2023.1195567)

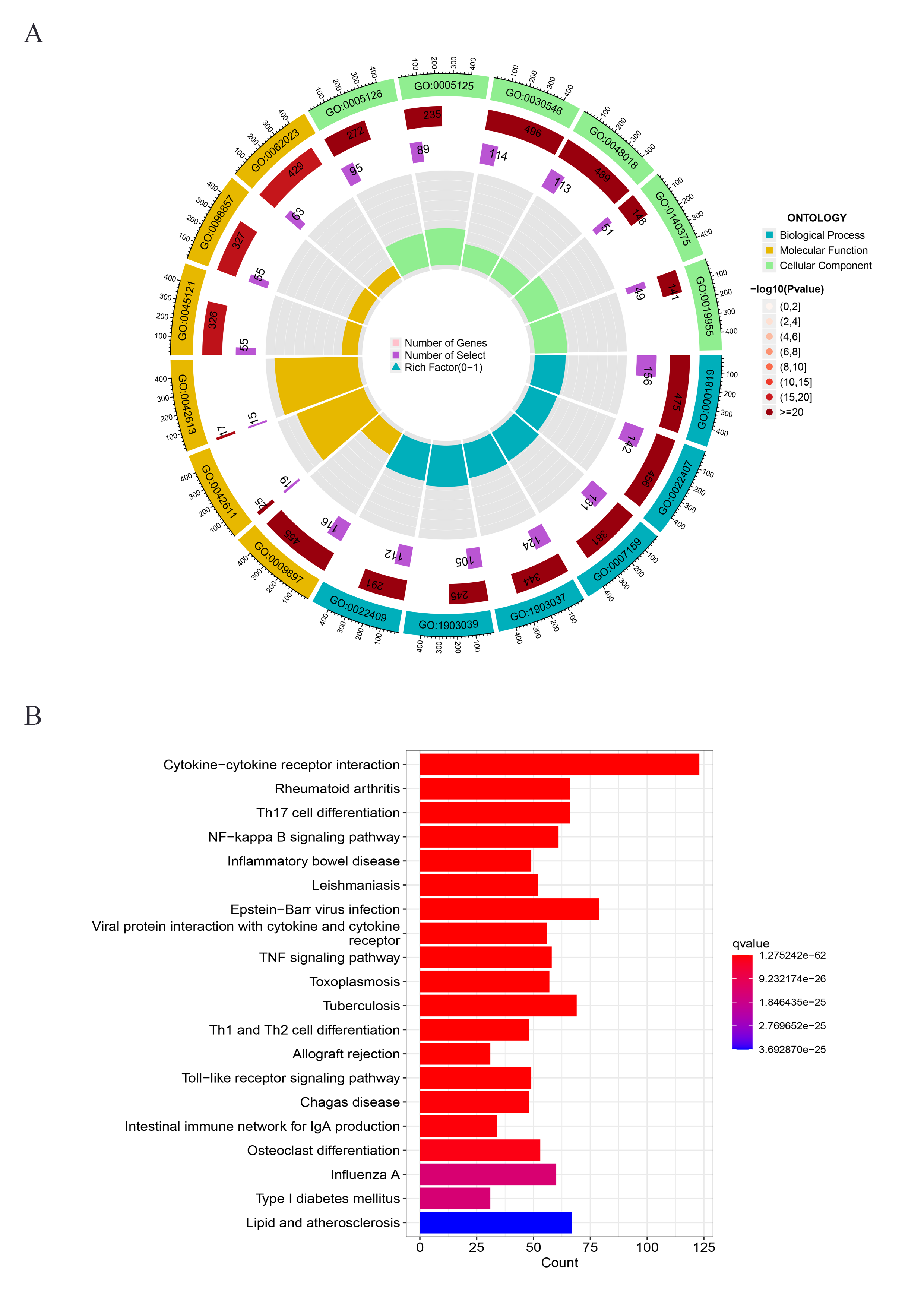

Supplement: Supplementary file 1 [file Image2.TIF]

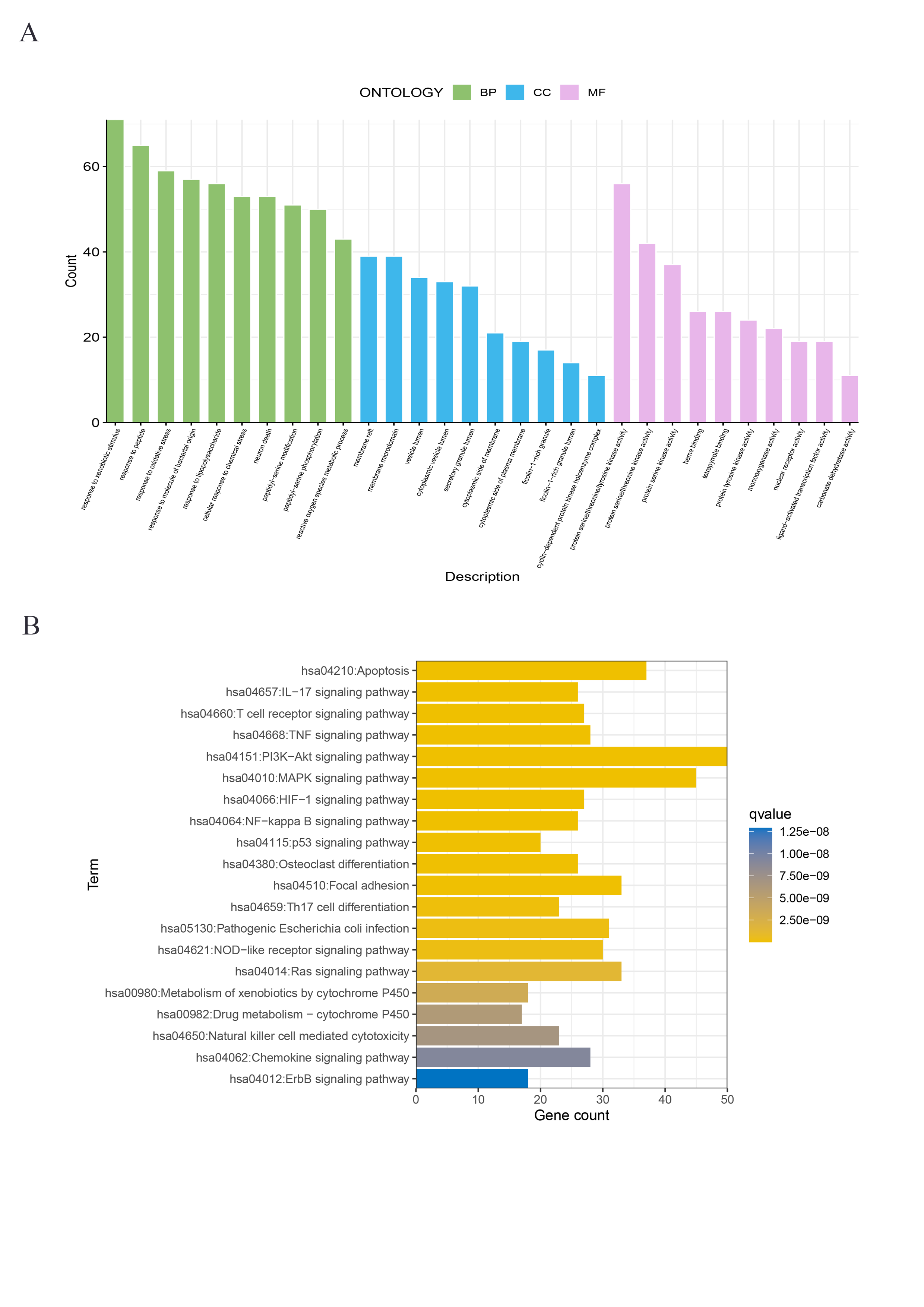

Supplement: Supplementary file 2 [file Image1.TIF]
